# Add‐on treatment with vinpocetine reduces seizure frequency and improves comorbidities in patients with loss‐of‐function γ‐aminobutyric acid type A receptor variants

**DOI:** 10.1002/epi.70310

**Published:** 2026-06-02

**Authors:** Cathrine E. Gjerulfsen, Vivian W. Y. Liao, Tomasz S. Mieszczanek, Anne V. Jakobsen, Elena Gardella, Kern Olofsson, Marina Nikanorova, Allan Bayat, Sebastian Ortiz, Sarah Weckhuysen, Cecilie Johannessen Landmark, Orrin Devinsky, Mary Chebib, Philip K. Ahring, Guido Rubboli, Rikke S. Møller

**Affiliations:** ^1^ Department of Epilepsy Genetics and Personalized Medicine Danish Epilepsy Centre, Filadelfia (member of European Reference Network EpiCARE) Dianalund Denmark; ^2^ Department of Regional Health Research, Faculty of Health Sciences University of Southern Denmark Odense Denmark; ^3^ Brain and Mind Centre, School of Medical Sciences, Faculty of Medicine and Health, University of Sydney Sydney New South Wales Australia; ^4^ Department of Child Neurology Danish Epilepsy Centre Dianalund Denmark; ^5^ Department of Interdisciplinary Health Research Danish Epilepsy Centre Dianalund Denmark; ^6^ Department of Psychology, Faculty of Health Sciences University of Southern Denmark Odense Denmark; ^7^ Department of Clinical Neurophysiology Danish Epilepsy Centre Dianalund Denmark; ^8^ Faculty of Health Sciences, University of Southern Denmark Odense Denmark; ^9^ Neurology Department University Hospital Antwerp Antwerp Belgium; ^10^ Translational Epilepsy Genomics Group, Vlaams Instituut voor Biotechnologie Center for Molecular Neurology, Vlaams Instituut voor Biotechnologie Antwerp Belgium; ^11^ Translational Neurosciences, Faculty of Medicine and Health Science, University of Antwerp Antwerp Belgium; ^12^ Department of Pharmacy Oslo Metropolitan University Oslo Norway; ^13^ National Center for Epilepsy, Oslo University Hospital (member of European Reference Network EpiCARE) Oslo Norway; ^14^ Department of Pharmacology Oslo University Hospital Oslo Norway; ^15^ Department of Neurology NYU Langone Medical Center New York New York USA; ^16^ Grossman School of Medicine, New York University New York New York USA; ^17^ Institute of Clinical Medicine, University of Copenhagen Copenhagen Denmark

**Keywords:** Cavinton, comorbidities, developmental and epileptic encephalopathies (DEE), precision medicine

## Abstract

**Objective:**

The semisynthetic compound vinpocetine has gained attention as a potential precision medicine for developmental and epileptic encephalopathies caused by loss‐of‐function (LoF) variants in γ‐aminobutyric acid type A (GABA_A_) receptor genes. As a positive allosteric modulator of GABA_A_ receptors, case reports suggest that vinpocetine can reduce epileptiform activity and seizure frequency, while improving cognitive function in patients with GABA_A_ receptor‐related epilepsies. Here, we extend these observations with a retrospective observational study evaluating the response to vinpocetine in an additional seven patients.

**Methods:**

Patients initiated treatment with vinpocetine between 2018 and 2025 at the Danish Epilepsy Centre or abroad. Clinical data were collected from medical records, seizure diaries, and neuropsychological assessments. The modulatory efficacy of vinpocetine was investigated using electrophysiological studies.

**Results:**

Nine patients harboring eight GABA_A_ receptor LoF variants were given add‐on vinpocetine treatment. Electrophysiological analyses confirmed dose‐dependent positive modulation by vinpocetine across tested variants. Six patients with a median age of 15.5 years (range = 6–29) continued treatment for a median of 24 months (range = 12–90), whereas three discontinued due to adverse effects (AEs) or lack of efficacy. The patients' level of function ranged from normal to moderate intellectual disability, psychiatric comorbidities, and behavioral disturbances. Four patients initiated vinpocetine due to uncontrolled seizures. One became seizure‐free, and two experienced a 50%–55% reduction. Electroencephalograms demonstrated improved spike–wave indexes in four patients. Six showed improvement in nonseizure factors, and caregivers reported reduced aggressivity and better vocabulary in one. Vinpocetine was well tolerated, with only mild and reversible AEs reported.

**Significance:**

Adjunctive vinpocetine shows promise as a targeted therapy for patients with GABA_A_ receptor LoF variants, decreasing seizure frequency and positively impacting nonseizure factors, with only mild AEs reported. Vinpocetine may be a safe and effective therapy for patients with GABA_A_ receptor‐related epilepsies, which should be investigated further in future *N*‐of‐1 trials.

**Key points**

- Vinpocetine shows promise for patients with loss‐of‐function GABA_A_ receptor variants, reducing seizure frequency and improving neurodevelopmental comorbidities in real‐world clinical settings.
- Among patients with uncontrolled epilepsy, 75% achieved a 50%–100% reduction in seizure frequency, with accompanying improvements in spike–wave indices on follow‐up EEGs.
- Improvements in adaptive behavior, executive function, and quality of life emerged within the first 6 months of treatment.
- Electrophysiological studies demonstrated dose‐dependent positive allosteric modulation of all tested GABA_A_ receptor variants by vinpocetine, including variants with reduced GABA potency.
- Regarding patient stratification, functional characterization of GABA_A_ receptor variants is critical for identifying likely responders, although interindividual variability limits precise outcome prediction.

## 1 INTRODUCTION

γ‐Aminobutyric acid type A (GABA_A_) receptors are the major inhibitory ion channels of the mammalian brain. Missense variants in genes encoding several GABA_A_ receptor subunits (α1–a5, β1–3, γ2, δ) can cause epilepsy and neurodevelopmental disorders (e.g., intellectual disability [ID], autism spectrum disorder [ASD], and attention‐deficit/hyperactivity disorder [ADHD]).^1^, ^2^, ^3^, ^4^, ^5^, ^6^, ^7^ Although patients with GABA_A_ receptor loss‐of‐function (LoF) variants often have relatively mild phenotypes, many remain refractory to antiseizure medication (ASM).^1^, ^3^, ^4^, ^5^, ^7^ Others may not experience seizures but present with neurodevelopmental delay for which treatment options are limited.

Vinpocetine, a semisynthetic derivative of vincamine, was launched in 1977 to treat cerebrovascular disorders, and later tried for multiple neurological conditions.^8^ Details about vinpocetine as an anticonvulsant were first reported in 1986^9^ and confirmed by studies reporting that vinpocetine suppressed epileptic cortical activities more effectively than some ASMs.^10^ Trials have later investigated the efficacy of vinpocetine in patients diagnosed with different epilepsy type classifications, with various results.^9^, ^11^, ^12^

Vinpocetine as a potential precision medicine for epilepsy caused by LoF GABA_A_ receptor variants emerged in 2019. This case study demonstrated that add‐on vinpocetine dramatically reduced epileptiform activities and improved the Clinical Global Impression (CGI) rating in a patient with Lennox–Gastaut syndrome caused by an LoF missense variant (Tyr302Cys) in *GABRB3*.^13^ Additionally, results of add‐on vinpocetine in a patient with an LoF variant (Arg112Gln) in *GABRA1* resulted in seizure freedom and marked improvements in social behavior and cognitive functions.^14^ Pharmacological analysis confirmed that vinpocetine is a positive allosteric modulator of GABA_A_ receptors, potentially offsetting the functional consequences of LoF GABA_A_ receptor variants. Later, a case report described how a patient with a *GABRG2* variant improved with vinpocetine add‐on therapy, although this variant was not functionally characterized.^15^

To assess the possibility of vinpocetine being a precision medicine, we conducted an observational, retrospective study investigating the clinical response to adjunctive vinpocetine treatment in patients with LoF GABA_A_ receptor variants. The evaluated patients were treated as part of daily clinical practice and had varying degrees of neurodevelopmental impairment with or without intractable epilepsy.

We present data from six patients treated with vinpocetine at the Danish Epilepsy Centre, Filadelfia, Dianalund, Denmark, and three additional patients treated at collaborating centers. Two patients represent follow‐up studies from Billakota et al.^13^ and Gjerulfsen et al.^14^ Furthermore, we performed electrophysiological assessment of vinpocetine on the harbored GABA_A_ receptor variants.

## 2 MATERIALS AND METHODS

### 2.1 Study design and ethics

At the Danish Epilepsy Centre, patients with GABA_A_ receptor LoF variants initiated treatment with vinpocetine as a part of daily clinical practice by the treating physician. Thus, vinpocetine was not initiated as a part of a clinical trial. The study was approved by the institutional review board at the Danish Epilepsy Centre (IRB EMN‐2024‐01998), and informed consent was obtained for all the included patients by their caregivers. Data in this observational study were collected retrospectively from the patients' electronic medical records and seizure diaries to investigate the response on seizure frequency and cognitive abilities. As vinpocetine, in the participating countries, is considered a dietary supplement, no further approvals were needed. Data from patients treated with vinpocetine abroad were retrospectively collected using a deidentified data sheet completed by the treating physician. Patients from abroad were treated at centers in the United States, Uruguay, and Belgium; thus, the examinations to estimate treatment outcomes could vary, as well as the vinpocetine preparation prescribed.

Updates on treatment details and vinpocetine response were collected for two of the three previously published cases.^13^, ^14^

### 2.2 Treatment procedures at the Danish Epilepsy Centre

Before initiation of vinpocetine, patients had normal electrocardiograms, pulse, blood pressure, hematological parameters, liver enzymes, kidney function, and electrolyte levels. Serum concentrations of ASMs were measured before initiation of vinpocetine to detect possible drug–drug interactions as part of the routine follow‐up by liquid chromatography coupled with tandem mass spectrometry analyses. To ensure safety and evaluate response to vinpocetine, examinations were performed at baseline and regularly during treatment. Electroencephalography (EEG) was performed at baseline and every 6 months of treatment, irrespective of seizure control. The spike–wave index (SWI) was evaluated on routine awake and drowsy EEG and calculated through visual inspection by the same EEG expert, following a standardized protocol. Interictal epileptiform activity was classified according to the criteria proposed by the International Federation of Clinical Neurophysiology and validated.^16^ The potential changes in the rate of interictal epileptiform discharges on EEGs were quantified as an indirect measure of treatment effect and not because of suspension of developmental and/or epileptic encephalopathy with spike–wave activation in sleep. A neuropsychological assessment was performed at baseline and after 6 and 12 months of treatment and included the following:

- Assessment of cognition by an age‐appropriate test (i.e., Bayley‐IV, Wechsler Preschool and Primary Scale of Intelligence, Wechsler Intelligence Scale for Children, or Wechsler Adult Intelligence Scale) based on the patient's perceived cognitive versus biological age. Level of cognitive abilities was classified as follows: (1) mild ID, with intelligence quotient (IQ) at 50–69; (2) moderate ID, with IQ at 35–49; and (3) severe ID, with IQ at 20–34.
- Assessment of the patients' adaptive behavior by the Vineland Adaptive Behavior Scales, Third Edition or the Adaptive Behavior Assessment System, Third Edition. Scores were evaluated according to guidelines, by which low scores (< 70) represent greater difficulties in adaptive behavior.^17^
- Assessment of executive functions by Behavior Rating Inventory of Executive Function, Second Edition (patients aged 5–18 years) or Behavior Rating Inventory of Executive Function, Preschool Version (patients aged 2–5 years). T‐scores ≥ 65 are considered to represent difficulties of clinical relevance.^18^, ^19^
- Assessment of quality of life by Quality of Life in Childhood Epilepsy Questionnaire‐55 (QOLCE‐55), in which a higher score represents better quality of life.^20^

### 2.3 Data collection and outcome measures

Data collection included patient demographics and clinical data related to seizures and comorbidities. For patients with ongoing seizures, the primary outcome was a change in monthly seizure frequency registered in the patients' electronic seizure diaries. The average number of seizures 3 months before vinpocetine initiation determined the baseline seizure frequency. Seizure types were defined using International League Against Epilepsy criteria.^21^ For the patients with seizures controlled, the primary outcome was changes in comorbidities evaluated by a neuropsychological assessment at baseline and after 6 and 12 months of treatment. We also assessed adverse effects (AEs) related to vinpocetine, serum concentrations of ASMs, and reasons for discontinuation of vinpocetine.

### 2.4 Vinpocetine preparation

To ensure the quality of the product used for treatment, vinpocetine was bought as a magistral preparation in capsules for all patients treated at the Danish Epilepsy Center. The maximum dose was ~1 mg/kg/day. Vinpocetine was initiated with one third of the maximum dose administered before bedtime to decrease potential AEs. Patients were titrated by one third of the maximum dose every third week until the maximum dose was reached. Vinpocetine was taken with food three times per day to increase bioavailability.^22^

Patients treated abroad bought vinpocetine from shops selling nutritional supplements, as magistral preparation of vinpocetine was not available. Thus, brands and the composition of the vinpocetine supplements varied.

### 2.5 Vinpocetine activity on variant GABA_A_ receptors

**FIGURE 1:**
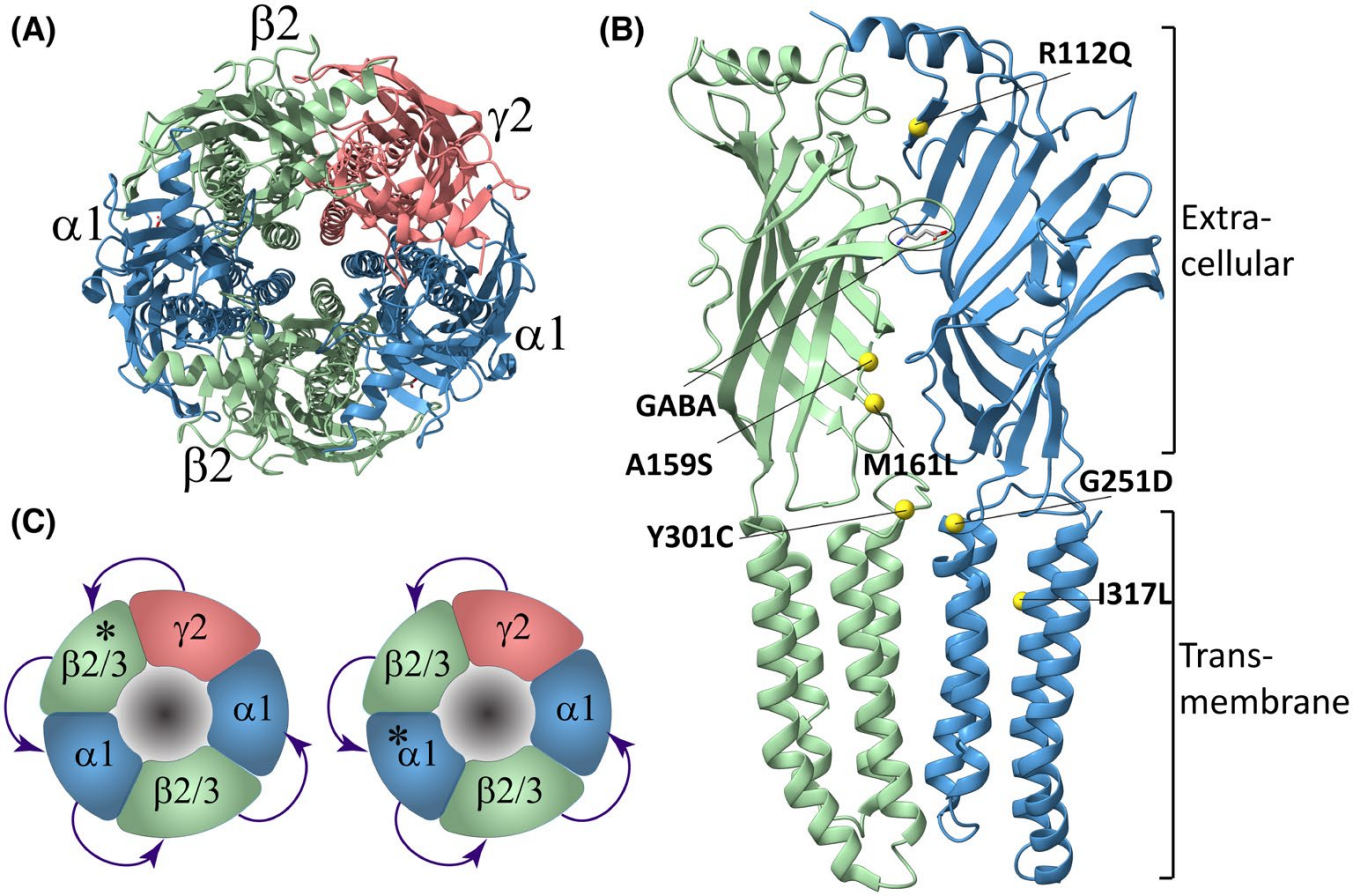
γ‐Aminobutyric acid type A (GABA_A_) receptor structure, location of missense variants, and experimental design. (A) Top‐down view of the cryoEM structure of the human α1β2γ2 GABA_A_ receptor (pdb: 6X3Z) visualized using UCSF ChimeraX.^24^ Receptor subunits are colored as follows: α1, blue; β2, green; γ2, coral, highlighting the pentameric arrangement. (B) Side view of a β2/α1 subunit interface showing GABA (stick representation) and the three‐dimensional location of the missense variants of interest (yellow spheres). *GABRB3* Y302C is a paralogous variant of the *GABRB2* Y301C, hence is found in the equivalent location in the β3 subunit. (C) Concatenated receptor construct design used to assess vinpocetine activities. Missense variants were introduced into a single subunit (indicated by asterisks). The left graphic shows the construct used to study *GABRB2* or *GABRB3* variants and the right to study *GABRA1* variants. Subunits were linked via short peptide linkers (purple arrows) to fix receptor stoichiometry, ensuring the expressed receptors only contain one mutant subunit, reflecting the main mutant receptor population of the heterozygous individuals carrying them.

GABA_A_ receptors are heteropentameric ion channels most commonly composed of two α1, two β2 or β3, and one γ2 subunit, arranged in a defined manner and exemplified by the top‐view structure of the α1β2γ2 receptor (Figure 1A). Pathogenic missense variants shown on the side‐view structures β2 and α1 (Figure 1B) were individually introduced into a single subunit of concatenated GABA_A_ receptor cDNAs: γ2‐β2*‐α1‐β2‐α1 for *GABRB2* variants (where * denotes the mutant subunit) or γ2‐β3*‐α1‐β3‐α1 for *GABRB3* and γ2‐β3‐α1*‐β3‐α1 *GABRA1* variants (Figure 1C), using methods well described in previous publications.^1^, ^5^, ^6^, ^23^ The concatenated cDNA receptor constructs were then expressed in *Xenopus laevis* oocytes. Vinpocetine activity was assessed by coapplying increasing concentrations of vinpocetine with a fixed submaximal half maximal effective concentration (EC_10_) concentration of GABA, a concentration that elicits 10% of the maximal response to GABA (10 mmol·L^−1^), of both wild‐type (WT) and mutant receptors. To determine vinpocetine modulatory potency, the EC_50_ value–concentration that elicits 50% of the maximal response to vinpocetine was calculated by fitting the modulatory response to the Hill equation to generate concentration–response curves. Modulatory responses were calculated as the percentage increase in peak current amplitude elicited by coapplication of vinpocetine and EC_10_ GABA, relative to the response evoked by EC_10_ GABA alone. Maximum efficacy (E_max_) is the maximum modulatory (%) effect observed when a concentration of vinpocetine is coapplied with GABA EC_10_ (Supporting Information).

### 2.6 Statistical analysis

To compare vinpocetine activity (EC_50_ and E_max_ values) on GABA_A_ receptor variants, one‐way analysis of variance followed by Dunnett post hoc test was used for multiple comparisons. Analyses were performed using GraphPad Prism version 10. A *p*‐value < .01 was considered statistically significant. Data are presented as mean ± SD. Due to the small sample size of patients, only descriptive analysis of clinical data was performed. To analyze serum concentrations of the administered ASMs and possible drug–drug interactions, concentration–dose (C/D) ratios and percentual change from baseline to follow‐up were calculated.

## 3 RESULTS

Nine patients (three females, six males) treated with vinpocetine were evaluated in this retrospective observational study; six (#1–#6) at the Danish Epilepsy Centre, and the remaining three at sites in Belgium (#7), the United States (#8), and Uruguay (#9). Treatment outcomes of Patients #1 and #8 were published previously.^13^, ^14^ We updated their treatments and outcomes at last follow‐up, 4 and 7 years after initiation of vinpocetine. Table 1 summarizes the results. Patients initiated treatment between April 2018 and January 2025. Thus, we present outcome assessments up to 7 years on vinpocetine treatment.

**TABLE 1:** Vinpocetine treatment and epilepsy outcome measures.

Patient #/publication	Gender/variant	VNP initiated (age)	Sz types/overall frequency at baseline	Treatment duration at last follow‐up	VNP maintenance dose/preparation	ASMs at last follow‐up (other)	Sz reduction at last follow‐up	Interictal EEG changes from baseline to last follow‐up	Adverse effects
#1/^14^	M/*GABRA1*, c.335G>A p.(Arg112Gln), de novo	August 2021 (16 years)	Myoclonic Sz, FPC, GTC/weekly Sz	5 years 5 months	70 mg (.89 mg/kg/day)/magistral	BRV, VPA, VNS (sertraline)	100%	Reduction in SWI from .78% in two foci at baseline to <.11% after 12 months of treatment	Headache (resolved)
#2	M/*GABRA1*, c.752G>A, p.(Gly251Asp), de novo	November 2023 (12 years)	Yearly Sz	8 months (discontinued)	30 mg (.88 mg/kg/day)/magistral	LEV, ZNS	‐	‐	Headache, tinnitus
#3	M/*GABRB2*, c.902A>G, p.(Tyr301Cys), de novo	December 2023 (17 years)	Eyelid myoclonia/56 Sz per month	2 years 1 month	60 mg (.88 mg/kg/day)/magistral	TPM, BRV (risperidone, atomoxetine)	55%	‐	None
#4	M/*GABRA1*, c.752G>A, p.(Gly251Asp), de novo	January 2024 (17 years)	Sz‐free	2 years	50 mg (.96 mg/kg/day)/magistral	LEV	‐	Reduction in SWI from 1.22% in three foci at baseline to .11% in two foci after 6 months of treatment, and <1% after 18 months	None
#5	M/*GABRA1*, c.949A>C p.(Ile317Leu), de novo	June 2024 (6 years)	Sz‐free	1 year 7 months	30 mg (.86 mg/kg/day)/magistral	VPA, BRV	‐	EEG was normal at baseline and at 6‐ and 12‐month follow‐up	None
#6	F/*GABRB2*, c.481A>C, p.(Met161Leu), de novo	January 2025 (15 years)	GTC, FIC/2 Sz per month	1 year	60 mg (1 mg/kg/day)/magistral	LTG, LEV	50%	Reduction in SWI from 1.11% in the frontotemporocentral area at baseline to .44% only during hyperventilation after 6 months of treatment	None
#7	F/*GABRB2*, c.475G>T p.(Ala159Ser)	April 2024 (26 years)	Yearly Sz	6 months (discontinued)	30 mg (.55 mg/kg/day)/nonmagistral	LTG, VPA, CLN (methylphenidate)	‐	‐	Increased impulsivity, irritability; headache
#8/^13^	F/*GABRB3*, c.905A>G, p.(Tyr302Cys)	April 2018 (29 years)	Sz‐free	7 years 8 months	60 mg (NA)/nonmagistral	TPM, FBM (sertraline)	‐	SWI declined from 5.0% to 2.9% after 6 months of treatment	None
#9	F/*GABRA1*, *c*.641G>A p.(Arg214His)	June 2023 (7 years)	GTC, FIC/weekly Sz	6 months (discontinued)	60 mg (2.4 mg/kg/day)/nonmagistral	LTG, ketogenic diet	0%	‐	Headache

*Note*: Mild ID when IQ is estimated to be 69–50, moderate ID when IQ 49–35, severe ID when IQ 34–20.

Abbreviations: ASM, antiseizure medication; BRV, brivaracetam; CLN, clonazepam; EEG, electroencephalographic; F, Female; FBM, felbamate; FIC, focal impaired consciousness seizures; FPC, focal preserved consciousness seizures; GTC, generalized tonic–clonic seizures; ID, intellectual disability; IQ, intelligence quotient; LEV, levetiracetam; LTG, lamotrigine; M, male; NA, not available; SWI, spike–wave index; Sz, seizure(s); TPM, topiramate; VNP, vinpocetine; VNS, vagus nerve stimulator; VPA, valproic acid; ZNS, zonisamide.

### 3.1 Demographics and baseline characteristics

The nine patients had a median age of 15.5 years (range = 6–29 years) at the initiation of treatment with vinpocetine. All had LoF GABA_A_ receptor missense variants that were previously verified by electrophysiological assessment*s*,^1^, ^5^, ^6^ of which five of nine (56%) patients harbored variants of *GABRA1*, three of nine (33%) variants of *GABRB2*, and one of nine (11%) a variant of *GABRB3*. Two patients (#2 and #4) shared the same variant in *GABRA1* (c.752G>A, p.[Gly251Asp]). Seven of nine patients presented with ID classified as mild to moderate, and eight of nine (89%) with comorbidities (ASD, aggressivity, ADHD, anxiety, and obsessive–compulsive disorder). All patients suffered from epilepsy, with a median age at seizure onset of 9.5 months (range = 6–12 months). At the time of vinpocetine initiation, patients received a median of two (range = 1–3) antiseizure therapies, including vagus nerve stimulator and ketogenic diet. At baseline, three patients (#4, #5, #8) were seizure‐free, whereas the remaining six (#1, #2, #3, #6, #7, and #9) suffered from daily to monthly and yearly seizures. Seizure types are listed in Table 1. Patients with seizures controlled by conventional ASMs initiated treatment with vinpocetine to increase cognitive abilities and improve comorbidities.

### 3.2 Electrophysiological assessment of vinpocetine activity on variant receptors

The electrophysiologically assessed seven GABA_A_ receptor missense LoF variants are shown on the side‐view structures of β2 and α1 subunits (Figure 1). To investigate whether vinpocetine retained positive allosteric modulation at the variant receptors, electrophysiological assessments were performed before treatment in most cases (Patients #1–#6 and #9). For this, increasing concentrations of vinpocetine were coapplied with a fixed GABA EC_10_ (a concentration that elicits 10% of the maximal GABA response). The increase in current beyond that elicited by GABA EC_10_ alone is defined as a positive modulatory response and measured as a percentage increase of the EC_10_ response. The percentage modulatory response was plotted against increasing vinpocetine concentrations to determine the EC_50_ (potency) of vinpocetine, with the maximal modulatory effect defined as E_max_ (%).

**FIGURE 2:**
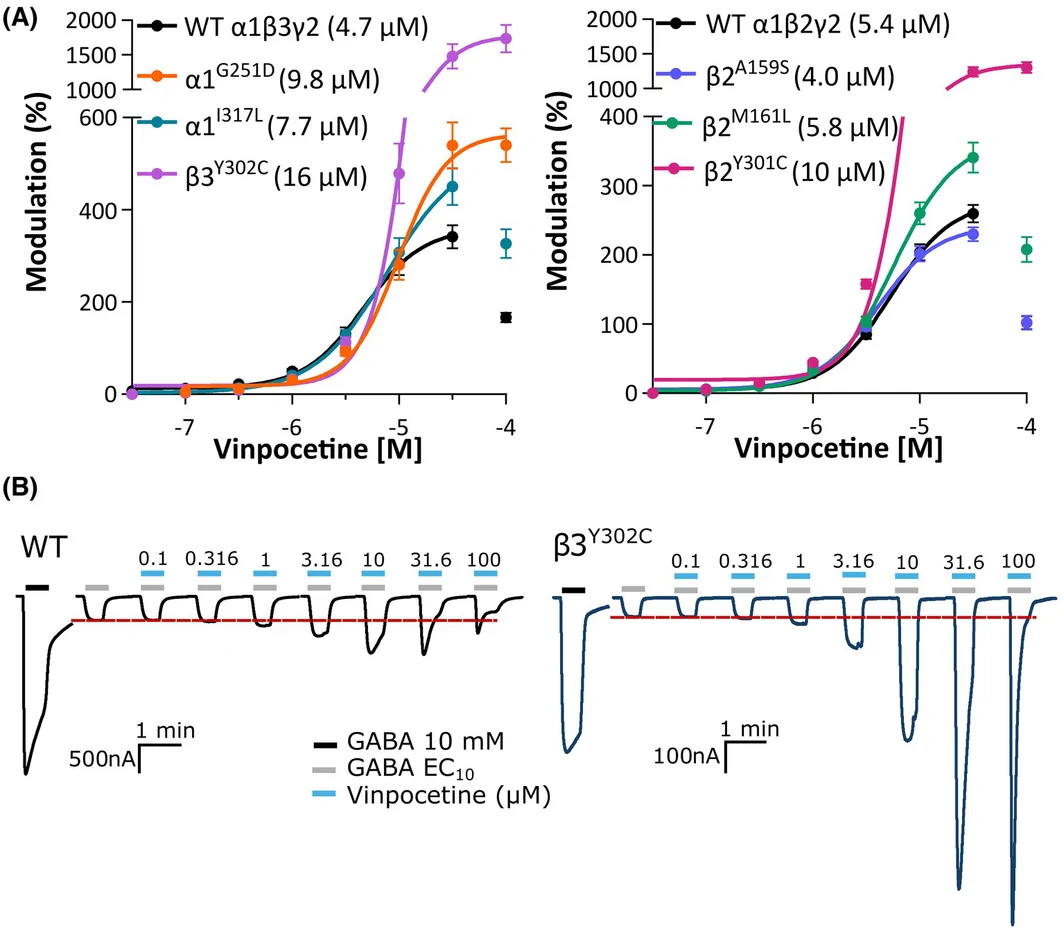
Modulation of wild‐type (WT) and variant containing γ‐aminobutyric acid type A (GABA_A_) receptors by vinpocetine. (A) Concentration–response curves for vinpocetine modulation of WT and mutant GABA_A_ receptors. Increasing concentrations of vinpocetine were coapplied with a fixed EC_10_ concentrations of GABA (see Tables S1 and S2) to WT and mutant receptors expressed in *Xenopus laevis* oocytes. Average modulatory responses ([I_vinpocetine_ − I_control_] / I_control_) are depicted as percent ± SEM as a function of the vinpocetine concentration for *n* ≥ 12 experiments across a minimum of two batches of oocytes. A Hill equation was fitted to the data using nonlinear regression. All variants were measured alongside their respective WT on the same day. *GABRA1* G251D, *GABRB2* Y301C, and *GABRB3* Y302C variants showed a two‐ to threefold reduction in vinpocetine potency compared to their respective WT receptors (see Table S3 for EC_50_ values). (B) Representative current traces showing vinpocetine concentration–responses for WT (black) and *GABRB3* Y302C (navy blue) receptors. The first response trace shows the maximal GABA response (10 mmol·L^−1^) used as a reference for maximal receptor activation by GABA. The second response corresponds to GABA EC_10_ alone, with the red dashed line marking this baseline response. Current amplitudes exceeding this line are indicative of vinpocetine‐mediated modulation. In WT receptors, increasing vinpocetine concentrations elicited progressively larger currents that plateaued below the maximal GABA response. In contrast, vinpocetine potentiation in *GABRB3* Y302C receptors exceeded the GABA maximal response.

Vinpocetine had similar potency and efficacy at the two WT receptors, α1β2γ2 and α1β3γ2, demonstrating similar actions across receptor complexes (Figure 2A). All mutant GABA_A_ receptors showed a dose‐dependent positive modulatory response to vinpocetine. The *GABRB2* variants, A159S (p.[Ala159Ser]), M161L (p.[Met161Leu]), along with *GABRA1* I317L (p.[Ile131Leu]), showed comparable potencies to their respective WT receptors. A modest two‐ to threefold reduction in vinpocetine potency was observed for receptors containing the variants *GABRA1* G251D (p.[Gly251Asp], *p* = .003), *GABRB2* Y301C (p.[Tyr301Cys], *p* < .001), and *GABRB3* Y302C (p.[Tyr302Cys], *p* < .001; Table 2).

**TABLE 2:** Results of neuropsychological assessments at baseline and during treatment with vinpocetine.

Patient # (age at BL)	ID status/comorbidities, BL	Cognitive functioning (WISC/WAIS‐IV/Bayley)			Adaptive behavior (VABS‐III or ABAS‐3)			Executive functions (BRIEF‐P/BRIEF‐2)			Subjective descriptions
		BL	6 months	12 months	BL	6 months	12 months	BL	6 months	12 months	
1 (16 years)	Normal/ASD, ADHD, anxiety, OCD, aggression	IQ: 73	‐	IQ: 80	‐	ABAS‐3: 75	ABAS‐3: 88	73	62	55	Improvement in OCD, anxiety, and depression; ADHD diagnosis withdrawn; overall improved quality of life
2 (12 years)	Mild ID/none	IQ: 44	‐	‐	VABS‐III: 78			‐	‐	‐	No improvements reported by caregivers
3 (17 years)	Moderate–severe ID/ASD, ADHD, aggression	‐	‐	‐	‐	‐	‐	‐	‐	‐	Increased vocabulary; less rigid; less behaviorally disturbed
4 (17 years)	Normal/ASD	IQ: 61	‐	IQ: 63	ABAS‐3: 71	ABAS‐3: 83	ABAS‐3: 85	59	50	50	Increased level of attention; behaved more calmly; less impulsiveness; more patience; more empathy
5 (6 years)	Moderate ID/ASD	RS: 108	RS: 104	RS: 105	VABS‐III: 55	VABS‐III: 59	VABS‐III: 58	55	48	58	More alert; increased vocabulary; better balance; more independent; increased concentration
6 (15 years)	Mild ID/ASD	IQ: 44	IQ: 46	IQ: 46	VABS‐III: 73	VABS‐III: 72	VABS‐III: 72	71	77	64	Better sleep; increased concentration
7 (26 years)	Mild ID/ADHD, behavioral issues, learning difficulties	‐	‐	‐	‐	‐	‐	‐	‐	‐	No improvements observed, stopped because of side effects after 2 months
8 (29 years)	Moderate ID/anxiety, OCD	‐	‐	‐	‐	‐	‐	‐	‐	‐	CGI score increased from 4 to 2.5; parents reported the patient to be “brighter,” more accurate, and engaged with tasks; increase in vocabulary
9 (7 years)	Moderate ID/ASD	‐	‐	‐	‐	‐	‐	‐	‐	‐	No improvements observed

*Note*: On VABS‐III, low scores (20–70) represent greater difficulties in adaptive behavior. On ABAS‐3, low scores (<70) represent greater difficulties in adaptive behavior. On BRIEF‐2, T‐scores ≥ 65 are considered to represent difficulties of clinical relevance. On BRIEF‐P, T‐scores ≥ 65 are considered to represent difficulties of clinical relevance. "‐" indicates test not performed.

Abbreviations: ABAS‐3, Adaptive Behavior Assessment System, Third Edition; ADHD, attention‐deficit/hyperactivity disorder; ASD, autism spectrum disorder; BL, baseline; BRIEF‐2, Behavior Rating Inventory of Executive Function, Second Edition (patients aged 5–18 years); BRIEF‐P, Behavior Rating Inventory of Executive Function, Preschool Version (patients aged 2–5 years); CGI, Clinical Global Impression; ID, intellectual disability; IQ, intelligence quotient; OCD, obsessive–compulsive disorder; RS, raw score; VABS‐III, Vineland Adaptive Behavior Scales, Third Edition; WAIS‐IV, Wechsler Adult Intelligence Scale, Fourth Edition; WISC, Wechsler Intelligence Scale for Children.

The E_max_ of vinpocetine in variants *GABRB2* A159S, M161L, and *GABRA*1 G251D, I317L were comparable to their respective WT (Supporting Information). In contrast, *GABRB2* Y301C and its paralogous *GABRB3* Y302C variant showed a four‐ to fivefold increase in E_max_ compared to WT (*p* < .0001). Both variants were found to be LoF in both GABA potency and GABA I_max_ (maximal peak current elicited by a saturating concetration of GABA).^1^, ^5^ Lin et al. demonstrated that the *GABRB3* Y302C variant reduced the receptor's maximum open probability to GABA.^25^ This indicates that GABA, even at saturating concentrations, fails to activate receptors with the *GABRB3* Y302C variant and likely also the paralogous *GABRB2* Y301C variant. Therefore, vinpocetine exhibited greater efficacy, enhancing receptor activation beyond that achieved by a saturating GABA concentration (10 mmol·L^−1^; Figure 2B).

### 3.3 Vinpocetine treatment and seizure‐related outcomes

Of the nine patients, three (33%) discontinued treatment due to AEs (#2 and #7) or lack of efficacy (#9). The remaining six (67%) are still treated with vinpocetine and have received treatment for a median period of 24 months (range = 12–90).

Four (#1, #3, #6, #9) patients initiated vinpocetine therapy for uncontrolled seizures. The median daily vinpocetine dose in those four was 60 mg (range = 50–70) added to different ASM combinations (Table 1). One (#9) was treated with a vinpocetine dose of 2.4 mg/kg/day, whereas the remaining three received <1 mg/kg/day. By adding vinpocetine to the existing ASM regimen, one patient became seizure‐free (Patient #1), and two experienced a 50%–55% reduction in seizures (#3, #6), whereas one did not experience any change in seizure frequency (#9). Of the two patients having yearly seizures at baseline (#2 and #7), neither experienced seizures during the 3‐ and 8‐month treatment periods before discontinuations of vinpocetine due to AEs.

**FIGURE 3:**
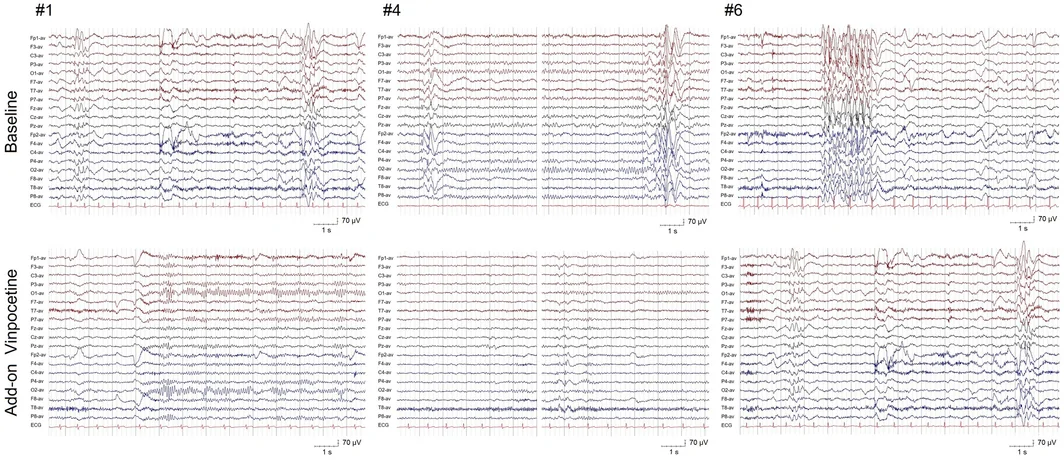
Interictal electroencephalographic (EEG) recordings in Patients #1, #4, and #6 before and after add‐on treatment with vinpocetine. In all patients, a reduction in the number of interictal epileptiform discharges (IEDs) and the number of epileptic foci was observed after 12, 6, and 6 months of add‐on vinpocetine treatment, respectively. In Patients #1 and #4, the interictal EEG was almost normalized during vinpocetine treatment (spike–wave index < .11% in both), whereas in Patient #6, discrete IEDs were observed only during hyperventilation.

EEG and neuropsychological assessments were performed every 6 months. Of the six patients treated at the Danish Epilepsy Centre, one (#3) was not able to participate in examinations investigating response to vinpocetine beyond seizure reduction, due to severe behavioral issues. One patient (#5) had a normal EEG at baseline and after 6 and 12 months of treatment. An improvement in SWI at follow‐up visits was observed in the remaining three patients (#1, #4, #6) treated at the Danish Epilepsy Centre (Table 1, Figure 3). An improvement in SWI on routine awake and drowsy EEGs was also observed in one patient treated abroad (#8). The remaining two patients treated at centers abroad, dosed with vinpocetine purchased as dietary supplements, did not report any beneficial effects related to vinpocetine (Figure 3).

### 3.4 Non‐seizure‐related outcomes

A neuropsychological assessment was performed at baseline and every 6 months of treatment, and included evaluation of cognitive abilities, adaptive behavior, executive function, and quality of life. Results are summarized in Table 2. Of the patients treated at the Danish Epilepsy Centre, five of six (83%) had an evaluation performed at baseline, and four of six (67%) were reevaluated after 6 and 12 months of treatment. One patient (#3) could not participate in the assessment, and one (#2) stopped treatment due to AEs.

Cognitive abilities were evaluated by either the Bayley‐IV or the Wechsler scales. In three of the four patients evaluated more than once (#1, #4, #5, #6), the estimated IQs at last follow‐up were the same as baseline or slightly higher, indicating an expected development relative to the patient's level of function. Thus, cognitive abilities developed despite the underlying genetic disorder, and treatment with ASMs, which can negatively affect cognitive abilities in patients.^26^ The fourth patient (#5) remained at the same raw score level at follow‐up, indicating a stagnation in cognitive abilities. However, qualitative observations indicated improvements in alertness and concentration, which are fundamental to cognitive functioning. Scores from follow‐up evaluations of adaptive behavior were available in four patients and revealed better scores in two of four (#1 and #4) and at the same standardized level in the other two patients (#5, #6). This indicates an accelerated or expected development, respectively, in all four patients. Executive functions were estimated to be the same in one of four (25%; #5) and improved in three of four (75%; #1, #4, #6) patients. The greatest increase in adaptive behavior and executive functioning was observed at the assessment 6 months after initiation of vinpocetine. Only Patient #6 did not improve after 6 months of treatment. Valproic acid was introduced shortly before the initiation of vinpocetine in this patient, and significant AEs were reported. This may have masked any potential effect of vinpocetine at the 6‐month follow‐up examination and explains why the improvements were first detectable after 12 months of treatment.

Quality of life was investigated for two patients (#5 and #6) and revealed an increase in scores of QOLCE‐55 from 57 to 62 and from 30 to 52, respectively. The higher scores represent a higher quality of life reported by the caregiver, despite cognitive abilities, adaptive behavior, and executive function, which remained at the same level or improved only slightly. Patient #3 initiated vinpocetine at his institution due to severe behavioral disturbances and was therefore not evaluated by a neuropsychologist. After 6 months of treatment, the caregivers of the patient reported less rigid behavior, fewer aggressive episodes, and improved vocabulary. Before treatment with vinpocetine, he was unable to participate in consultations with his treating clinicians or have his blood pressure measured. However, this has been possible after vinpocetine was initiated.

CGI, rating the severity of the illness, was estimated for Patient #8 by caregivers blinded to therapy. Their score improved from 4 to 2.5, consistent with better function after vinpocetine was added.

### 3.5 Tolerability and safety

Add‐on treatment with vinpocetine was generally well tolerated. Observed AEs paralleled literature reports.^9^, ^11^, ^12^, ^14^ Three patients (#1, #2, #7) experienced AEs, consisting of headache, tinnitus, impulsivity, and irritability. The headache was resolved with a dose reduction of 10 mg vinpocetine in Patient #1. Treatments were stopped in two patients (#2, #7) due to the AEs after 8 (#2) and 2 (#7) months of treatment, consisting of tinnitus, headache, increased impulsivity, and irritability.

To ensure safety, neurological evaluation, blood test, and electrocardiography were performed for the patients treated at the Danish Epilepsy Centre. No abnormalities were observed. Serum concentrations were available for five of six (83.3%) of the patients treated at the Danish Epilepsy Centre. Reflecting the real‐world conditions of this observational study, the existing ASM regimen in the treated patients was not held constant during treatment with vinpocetine. Therefore, C/D ratios and percentage changes from baseline to follow‐up were calculated. Results are summarized in Table 3 and illustrate a tendency toward a decrease in C/D ratios for several of the coadministered ASMs.

**TABLE 3:** ASM C/D ratio before and after add‐on vinpocetine.

Patient #	Baseline			6‐month follow‐up				12‐month follow‐up			
	ASM dose, mg/day	Serum concentration, μmol·L^−1^	C/D ratio	ASM dose, mg/day	Serum concentration, μmol·L^−1^	C/D ratio	Change in ratio since baseline, %	ASM dose, mg/day	Serum concentration, μmol·L^−1^	C/D ratio	Change in ratio since baseline, %
#1	VPA: 300 mg BRV: 235 mg	p‐VPA: 169 p‐BRV: 3.7	VPA: .563 BRV: 1.016	VPA: 450 mg BRV: 235 mg VNP: 60 mg	p‐VPA: 220 p‐BRV: 3.0	VPA: .489 BRV: .013	VPA: 86.79% BRV: 81.08%	VPA: 450 mg BRV: 235 mg VNP: 70 mg	p‐VPA: 160 p‐BRV: 3.5	VPA: .356 BRV: .015	VPA: 63.12% BRV: 94.59%
#2	LEV: 1200 mg ZNS: 100 mg	p‐LEV: 76 p‐ZNS: 41	LEV: .063 ZNS: .41	LEV: 1200 mg ZNS: 125 mg VNP: 30 mg	p‐LEV: 64 p‐ZNS: 53	LEV: .053 ZNS: .424	LEV: 84.21% ZNS: 103.41%	‐		‐	‐
#3	TPM: 75 mg BRV: 200 mg	p‐TPM: 4.6 p‐ BRV: 19.3	TPM: .061 BRV: .097	TPM: 75 mg BRV: 200 mg VNP: 60 mg	‐	‐	‐	TPM: 75 mg BRV: 200 mg VNP: 60 mg ‐		‐	‐
#4	LEV: 500 mg	p‐LEV: 56	LEV: .112	LEV: 500 mg VNP: 50 mg	p‐LEV: 22	LEV: .044	LEV: 39.29%	LEV: 500 mg VNP: 50 mg	p‐LEV: 48	LEV: .096	85.71%
#5	VPA: 600 mg LEV: 1000 mg	p‐VPA: 316 p‐LEV: 34	VPA: .527 LEV: .034	VPA: 600 mg BRV: 100 mg VNP: 30 mg	p‐VPA: 300 p‐BRV: 3.4	VPA: .5 BRV: .034	VPA: 94.94%	VPA: 600 mg BRV: 100 mg VNP: 30 mg	p‐VPA: 381 p‐BRV: 5.7	VPA: .635 BRV: .057	VPA: 120.57% BRV: 167.65%
#6	LEV: 1500 mg	p‐LEV: 81	LEV: .054	LEV: 1500 mg VPA: 300 mg LTG: 50 mg VNP: 60 mg	p‐LEV: 48 p‐VPA: 140 p‐LTG: 19	LEV: .032 VPA: .47 LTG: .38	LEV: 59.26%	LEV: 1500 mg LTG: 500 mg VNP: 60 mg	p‐LEV: 95 p‐LTG: 37	LEV: .063 LTG: .074	LEV: 117.28% LTG: 19.47%

Abbreviations: ASM, antiseizure medication; BRV, brivaracetam; C/D, concentration–dose; LEV, levetiracetam; LTG, lamotrigine; p, plasma; TPM, topiramate; VNP, vinpocetine; VPA, valproic acid; ZNS, zonisamide.

## 4 DISCUSSION

Our clinical observations of nine patients treated with vinpocetine provide the most comprehensive data on GABA_A_‐modulating therapy for LoF GABA_A_‐related disorders.

Three of four patients with uncontrolled seizures experienced a marked (50%–100%) reduction in seizure frequency, and all four patients showed a reduction in epileptiform abnormalities. Six of nine patients experienced improvements, either qualitatively described by caregivers or quantified by neuropsychological assessments and caregiver‐reported questionnaires. Before vinpocetine treatment, one patient was too behaviorally disturbed to participate in assessments and evaluations. However, this patient had a 55% reduction in seizure frequency and fewer behavioral problems after 6 months of treatment. Some patients continue to benefit 6 years after vinpocetine was initiated.

The maximum dose of vinpocetine used for treatment at the Danish Epilepsy Centre was ~1 mg/kg/day. Studies suggest vinpocetine is well tolerated up to 1 mg/kg/day.^9^, ^11^, ^12^ However, vinpocetine 2 mg/kg/day has been used to treat pediatric patients with epilepsy.^11^ Some included patients may have experienced greater improvements if treated with higher doses of vinpocetine. Serum concentrations of the patients' existing treatment with ASMs were measured to evaluate possible drug–drug interactions, and a tendency toward decreased C/D ratio for several of the administered ASMs was observed, although the metabolism of these drugs differs. One patient had lamotrigine increased from 50 mg/day to 500 mg/day during treatment with vinpocetine. However, an increase in serum concentrations corresponding to this 10‐fold increase was not observed, which could indicate enzyme induction of UGT1A. It has been suggested in an in vitro study that vinpocetine may inhibit CYP3A4 and P‐glycoprotein, but this could not be confirmed in a clinical setting (Manda et al., 2015)^27^. In this study, specific pharmacokinetic interactions caused by add‐on vinpocetine were not possible to establish.

To broaden observations with vinpocetine treatment in patients with GABA_A_ receptor LoF variants, data from three patients treated abroad were included. One patient experienced a consistent response to treatment with a marked reduction in SWI and improved CGI, whereas the other two stopped treatment due to lack of efficacy and/or AEs. For all patients treated abroad, vinpocetine was acquired from shops selling nutritional supplements. Therefore, the amount of vinpocetine and other ingredients in the consumed preparations may have varied in contrast to the magistral preparation of vinpocetine used to treat Danish patients.

All variants in our cases were classified as loss/reduced GABA_A_ receptor function based on electrophysiological testing. In an earlier published case, vinpocetine was used in an 8‐year‐old boy with a *GABRG2* (c.254 T > A p.[Ile85Lys]); however, functional characterization of this variant was not performed at the time.^15^ Subsequent functional analysis demonstrated that this variant did not impair receptor function,^7^ suggesting this variant may not be the cause of the epilepsy.

Other randomized, blinded, or open‐label studies have evaluated vinpocetine as adjunctive therapy in unselected epilepsy cohorts to decrease seizure frequency^9^, ^11^ or improve cognitive outcomes.^12^ Although a decreased frequency of generalized tonic–clonic seizures was reported after vinpocetine, no significant increase in cognitive abilities was observed. In unselected epilepsy cohorts lacking functionally confirmed GABA_A_ receptor LoF variants, vinpocetine did not improve neuropsychiatric comorbidities or cognitive function.^9^, ^12^ Broader clinical benefits beyond seizure reduction thus appear more likely when vinpocetine is administered to patients with a verified LoF GABA_A_ receptor variant.

The included patients harbored variants in genes encoding GABA_A_ receptors. Two patients (#3, #8) carried paralogous variants (*GABRB3* p.[Tyr302Cys] and *GABRB2* p.[Tyr301Cys]), which showed a two‐ to threefold reduction in vinpocetine potency compared to WT receptors. Both variants decreased the maximum GABA‐evoked current caused by a reduction in GABA maximum open probability; thus, GABA alone was unable to elicit maximal receptor response even at high concentrations. Despite reduced potencies, vinpocetine enhanced the functional response of these two variant receptors. When coapplied with GABA, vinpocetine increased the inhibitory current beyond that achievable with GABA alone, this increase in modulatory efficacies compensate for its reduced potencies. The pharmacological profile of vinpocetine on these two paralogous variants may account for the favorable response observed.

In our cohort, two patients (#2, #4) carried the same variant (*GABRA1*, p.[Gly251Asp]). One improved in adaptive behavior and executive function, whereas the other discontinued vinpocetine due to AEs. Several factors may influence the response to vinpocetine, including concomitant medications and bioavailability, which may be 60%–100% higher when taken with food.^22^ Thus, interpersonal differences make it difficult to predict the outcome.

The study has limitations. Data were collected retrospectively, and neither patients nor caregivers were blinded to the intervention. A randomized, placebo‐controlled clinical trial is necessary to confirm the results. The sample was small, which did not allow calculation of significance. Additionally, patients treated abroad obtained vinpocetine from nutritional supplement shops, introducing potential issues with the quality and dosages used. Adherence to this supplementary treatment was assumed to be constant, as caregivers were responsible for the medication.

## 5 CONCLUSIONS

This retrospective observational study presents comprehensive data on patients with GABA_A_ receptor LoF variants treated with vinpocetine as a precision medicine. The findings suggest that vinpocetine can reduce seizure frequency, improve SWI, and enhance nonseizure outcome measures. Electrophysiological data support that vinpocetine can restore function in patients with GABA_A_ receptor LoF variants, indicating a potential compensatory mechanism. However, variability in dosing and product quality in patients treated abroad highlights the need to standardize preparations. Our results support that vinpocetine may be a promising targeted therapy for patients with GABA_A_ receptor LoF variants, especially when treatment is individualized and closely monitored. Controlled clinical trials are needed to validate these findings and establish optimal treatment protocols.

## AUTHOR CONTRIBUTIONS

*Conceptualization:* Cathrine E. Gjerulfsen, Vivian W. Y. Liao, Anne V. Jakobsen, Philip K. Ahring, Guido Rubboli, and Rikke S. Møller. *Data curation:* Cathrine E. Gjerulfsen, Tomasz S. Mieszczanek, Anne V. Jakobsen, Elena Gardella, Kern Olofsson, Marina Nikanorova, Allan Bayat, Sebastian Ortiz, Sarah Weckhuysen, Cecilie Johannessen Landmark, and Orrin Devinsky. *Writing—original draft:* Cathrine E. Gjerulfsen, Vivian W. Y. Liao, Anne V. Jakobsen, Cecilie Johannessen Landmark, Philip K. Ahring, Guido Rubboli, and Rikke S. Møller. *Functional testing:* Vivian W. Y. Liao, Philip K. Ahring, and Mary Chebib. *Writing—review and editing (equal):* Cathrine E. Gjerulfsen, Vivian W. Y. Liao, Tomasz S. Mieszczanek, Elena Gardella, Kern Olofsson, Marina Nikanorova, Allan Bayat, Sebastian Ortiz, Sarah Weckhuysen, Anne V. Jakobsen, Cecilie Johannessen Landmark, Orrin Devinsky, Mary Chebib, Philip K. Ahring, Guido Rubboli, and Rikke S. Møller.

## FUNDING INFORMATION

The Nan Family on behalf of Matilda partially funded this study (grant to V.W.Y.L. and P.K.A.). Additional support was provided by the Lundbeck Foundation (grant R383‐2022‐276 to R.S.M. and P.K.A.) and the Australian National Health & Medical Research Council (grant APP2019780 to P.K.A., M.C., V.W.Y.L., and R.S.M.).

## CONFLICT OF INTEREST STATEMENT

C.E.G. has received speaker honoraria from UCB Pharma and funding from Jazz Pharmaceuticals and UCB Pharma. C.J.L. has received speaker honoraria from Angelini, Eisai, Jazz Pharmaceuticals, and UCB Pharma. G.R. has received speaker fees from Angelini Pharma and UCB Pharma and has participated in advisory board meetings of Angelini Pharma and UCB Pharma. R.S.M. has received consulting fees from UCB, Orion, Saniona, and Immedica and speaker fees from Eisai, Angelini Pharma, Jazz Pharmaceuticals, Orion, and UCB. S.W. has received consulting and speaker fees from UCB, Xenon Pharmaceuticals, Lundbeck, Knopp Biosciences, Encoded Therapeutics, Eisai, Angelini Pharma, and Roche. The remaining authors have no conflict of interest. We confirm that we have read the Journal's position on issues involved in ethical publication and affirm that this report is consistent with those guidelines.

## Supporting information


**Table S1** γ‐Aminobutyric acid EC_10_ concentrations used for vinpocetine modulation experiments.
**Table S2** γ‐Aminobutyric acid EC_50_ values for wild‐type (WT) and variants showing they are all loss of function.
**Table S3** Parameters of vinpocetine activity from dose–response curves of wild‐type (WT) and variant receptors.

## Data Availability

The data that support the findings of this study are available from the corresponding author upon reasonable request.
