# Supplementary material for: Add‐on treatment with vinpocetine reduces seizure frequency and improves comorbidities in patients with loss‐of‐function γ‐aminobutyric acid type A receptor variants
Source: Epilepsia. 2026 Jun 2;67(8):4051–64. doi: 10.1002/epi.70310 (PMC13525584; doi:10.1002/epi.70310)
Supplement: Supplementary file 1 [file EPI-67-4051-s001.docx]

**Supplementary**

*Method - electrophysiology*

GABA was purchased from Merck (Darmstadt, Germany) and prepared as a 3.16 M stock solution in ultrapure water. Vinpocetine was obtained from Cayman Chemical (USA) and dissolved in DMSO to a final stock concentration of 15.8 mM, using gentle heating and vortexing to aid solubilisation. All working solutions were prepared to ensure a final DMSO concentration did not exceed 1%. HEPES monosodium salt was sourced from MP Biomedicals (Australasia), and all other chemicals were obtained from Merck (Darmstadt, Germany).

*Xenopus* oocytes obtained from Victor Chang Institute (Sydney, Australia) were injected with 25 ng / cell of cRNA of the WT and mutant concatenated receptors and were incubated for 2 days at 18 °C in ND 96 solution, (96 mM NaCl, 2 mM KCl, 1 mM MgCl_2_, 1.8 mM CaCl_2_, 5 mM HEPES monosodium, 2.5 mM sodium pyruvate, 0.5 mM theophylline, and 50 mg/L gentamicin; pH 7.4). Electrophysiological recordings of GABA-evoked responses in WT and mutant α1β2γ2 and α1β3γ2 receptors were conducted at room temperature using a custom two-electrode voltage clamp setup. Oocytes were voltage clamped at -60 mV with an Axon GeneClamp 500B amplifier. Currents were low-pass filtered at 20 Hz, digitised with a Digidata 1322B, and recorded at 200 Hz using pClamp 10.2 software. Episodic traces were collected in response to individual GABA/vinpocetine applications.

The GABA EC_10_ concentration (Table 1) was determined from the average GABA dose-response curves for each of the WT and variants. When this calculated concentration was applied to the oocytes, the experimental responses ranged from 7% to 15% of the maximal GABA-evoke current. The percentage efficacy max (E_max_) relative to GABA EC_10_ represents the maximal response modulated by vinpocetine, and was determined by the formula, Where I_max VPN_ the maximal measured current amplitude in the presence of the vinpocetine and GABA EC_10_ and I_GABA EC10_ is the current amplitude in the presence of GABA EC_10_ alone.

$$E_{max}=\left( \frac{I_{max VPN}{-I}_{GABA EC10}}{I_{GABA EC10}} \right)\times100$$

Concentration-response curves were generated in GraphPad Prism 10 by fitting the modulatory response at each vinpocetine concentration to the Hill equation for individual oocytes. Modulation was calculated as the percentage increase in peak current amplitude during co-application of vinpocetine with GABA (EC_10_), relative to the current evoked by GABA (EC_10_) alone:

$$Modulation=\left( \frac{I_{vinpocetine+GABA EC10}{-I}_{GABA EC10}}{I_{GABA EC10}} \right)\times100$$

Concentration–response data were fitted to the Hill equation:

$$Modulation=\frac{E_{max}\left( \left[ Vinpocetine \right] \right)^{nH}}{{[vinpocetine]}^{nH}+{{EC}_{50}}^{nH}}$$

EC_50_ represents the concentration of vinpocetine that produces half-maximal response and *nH* is the Hill slope.

**Table 1.** GABA EC_10_ concentrations used for vinpocetine modulation experiments.

| **WT/Variants** | **GABA EC_10_ (µM)** |
| --- | --- |
| **WT α1β2γ2** | 60 |
| β2^A159S^ | 135 |
| β2^M161L^ | 135 |
| β2^Y301C^ | 285 |
| **WT α1β3γ2** | 60 |
| α1^G251D^ | 175 |
| α1^I317L^ | 150 |
| β3^Y302C^ | 250 |

**Table 2.** GABA EC_50_ values for WT and variants showing they are all loss-of-function

| **WT/Variants** | **GABA EC_50_ (µM)** |
| --- | --- |
| **WT α1β2γ2** | 320^1^ |
| β2^A159S^ | 480^1^ |
| β2^M161L^ | 510^1^ |
| β2^Y301C^ | 950^1^ |
| **WT α1β3γ2** | 200^2^ |
| α1^G251D^ | 920^3^ |
| α1^I317L^ | 665^3^ |
| β3^Y302C^ | 1100^4^ |

**Table 3:** Parameters of vinpocetine activity from dose-response curves of wild type (WT) and variant receptors.

| **WT/Variants** | **Vinpocetine**  **EC_50_ (µM)** | **E_max_ (%)** | **Hillslope** | **N** |
| --- | --- | --- | --- | --- |
| **WT α1β2γ2** | 5.98 ± 2.2 | 259.6 ± 53.7 | 1.44 ± 0.18 | 18 |
| β2^A159S^ | 4.65 ± 2.4 | 229.9 ± 41.7 | 1.42 ± 0.25 | 17 |
| β2^M161L^ | 5.90 ± 2.2 | 340.7 ± 91.4 | 1.52 ± 0.17 | 18 |
| β2^Y301C^ | 10.9 ± 1.6 | 1327 ± 301 | 1.92 ± 0.33 | 15 |
| **WT α1β3γ2** | 5.35 ± 1.7 | 341.5 ± 100 | 1.32 ± 0.24 | 16 |
| α1^G251D^ | 10.6 ± 3.2 | 571.5 ± 153 | 1.65 ± 0.24 | 12 |
| α1^I317L^ | 8.99 ± 3.9 | 450.3 ± 153 | 1.27 ± 0.24 | 15 |
| β3^Y302C^ | 15.3 ± 3.9 | 1735 ± 749 | 2.16 ± 0.33 | 14 |

EC_50_, E_max_, and Hill slope values were derived from nonlinear regression fits of vinpocetine concentration-response. Data are shown as mean ± standard deviation (SD). N indicates the number of oocytes recorded across at least two batches.

**1.** Mohammadi, NA et al (2024) Distinct neurodevelopmental and epileptic phenotypes associated with gain- and loss-of-function *GABRB2* variants. *eBioMedicine*, **106**,105236. <https://doi.org/10.1016/j.ebiom.2024.105236>

**2.** Gjerulfsen, C.E et al (2023), Vinpocetine improved neuropsychiatric and epileptic outcomes in a patient with a *GABRA1* loss-of-function variant. *Ann Clin Transl Neurol*, **10**:1493-1498. <https://doi.org/10.1002/acn3.51838>

**3.** Musto, E et al (2024) GABRA1-related disorders: from genetic to functional pathways**.** Annals of Neurology **95(1)**, 27-41. <https://doi.org/10.1002/ana.26774>

**4**. Absalom, NL et al (2022). Gain-of-function and loss-of-function GABRB3 variants lead to distinct clinical phenotypes in patients with developmental and epileptic encephalopathies. *Nature Communication* **13**, 1822. <https://doi.org/10.1038/s41467-022-29280-x>
